# AI-Assisted Hiring Process and Older Workers: An Exploratory Study

**DOI:** 10.1093/geroni/igaa057.2260

**Published:** 2020-12-16

**Authors:** Moon Choi

**Affiliations:** KAIST, Daejeon, Taejon-jikhalsi, Republic of Korea

## Abstract

An increasing number of companies have adopted an artificial intelligence (AI) hiring system such as resume screening and/or AI-powered video interview. This study explored older adults’ attitudes toward AI and their intentions to apply for jobs based on AI-assisted hiring process. Data came from an online survey in South Korea (N=123). Results showed an acceptable reliability of the scale of attitudes toward AI (Cronbach’s a = 0.72) and indicated no statistically significant differences in the attitudes toward AI across age groups (F(4,118) = 0.41, p = .80). However, a one-year increase in age was associated with a 5.8% decrease in the likelihood of decisions to apply for jobs based on AI-assisted hiring process after accounting for attitudes toward AI as well as socioeconomic status. The findings raise a concern about older workers’ constraints in job mobility as well as early retirement due to technological change in the hiring process.

